# Highly stable carbon nanotube field emitters on small metal tips against electrical arcing

**DOI:** 10.1186/1556-276X-8-355

**Published:** 2013-08-16

**Authors:** Jun Mok Ha, Hyun Jin Kim, Hamid Saeed Raza, Sung Oh Cho

**Affiliations:** 1 Department of Nuclear and Quantum Engineering, Korea Advanced Institute of Science and Technology (KAIST), 373-1 Guseong, Yuseong, Daejeon 305-701, Republic of Korea

**Keywords:** CNT, Stability, Field emission, Arcing, Small metal tip, Metal mixture binder

## Abstract

Carbon nanotube (CNT) field emitters that exhibit extremely high stability against high-voltage arcing have been demonstrated. The CNT emitters were fabricated on a sharp copper tip substrate that produces a high electric field. A metal mixture composed of silver, copper, and indium micro- and nanoparticles was used as a binder to attach CNTs to the substrate. Due to the strong adhesion of the metal mixture, CNTs were not detached from the substrate even after many intense arcing events. Through electrical conditioning of the as-prepared CNT emitters, vertically standing CNTs with almost the same heights were formed on the substrate surface and most of loosely bound impurities were removed from the substrate. Consequently, no arcing was observed during the normal operation of the CNT emitters and the emission current remained constant even after intentionally inducing arcing at current densities up to 70 mA/cm^2^.

## Background

Carbon nanotubes (CNTs) are widely used as field emission electron emitters for X-ray tubes [1-4], field emission displays [5], and high-resolution electron beam instruments [6,7] because of their excellent electron emission property, chemical inertness, and high electrical and thermal conductivity [8,9]. In spite of these superior characteristics, practical applications of CNT field emitters to devices particularly requiring high-voltage operation are limited due to unstable electron emission properties of the CNT emitters. Electron beam current emitted from CNT emitters can be fluctuated or degraded because CNTs are damaged by the back bombardment of ions produced from the residual gas [10,11] or CNTs are structurally deformed due to excessive Joule heating [12,13]. More seriously, emission current can be abruptly dropped because CNTs are detached from a substrate [14]. If a very high current (300 nA per single CNT) flows through a CNT, adhesion between the CNT and the substrate becomes weak due to resistive heating and accordingly the CNT can be peeled off from the substrate [14,15], or a strong electric field exerts electrostatic force on CNTs, leading to the detachment of the CNTs [15,16]. Weak adhesion of CNTs to a substrate deteriorates the removal of CNTs.

In addition, if CNT emitters are operated at a high voltage or at a high electric field, electrical arcing (or vacuum breakdown) can occur. Arcing can be initiated by the removed CNTs [17], impurities on the CNTs or substrates [18,19], protrusion of CNTs [10], low operating vacuum [10], and a very high electric field [20-23]. Since arcing is accompanied with a very high current flow and it can produce a plasma channel near the emitter, CNTs are seriously damaged or sometimes CNTs are almost completely removed from the substrate by the arcing events [17,20]. Detachment of CNTs from a substrate is an irreversible catastrophic phenomenon for a device operation [14]. In addition to the detachment of CNTs, arcing induces a sudden voltage drop, and thus, device operation is stopped. Therefore, for a stable operation of a device using CNT emitters, arcing should be prevented. Particularly, CNT emitters on small metal tips (diameter < 1 mm) are necessary for miniature X-ray tubes [1-4] and micro-focus X-ray tubes [6,7]. Small metal tips produce much higher electric field than flat substrates at the same applied voltage due to their sharp geometry. As a consequence, CNT emitters on small metal tips can suffer from much serious and frequent arcing, and hence, stable operation of the CNT emitters against arcing is a big issue [4,14].

So far, few papers have been reported on CNT emitters to withstand arcing, although some methods to reduce arcing events have been reported, including the operation of the CNT emitters under ultrahigh vacuum (approximately 10^−9^ Pa) [24,25], plasma treatment of the emitters [10,26], and removal of organic impurities by firing [19]. Here, we present an approach to fabricate CNT emitters on small metal tips that show extremely high stability against arcing. Using a metal alloy as a binder, CNT emitters can be strongly attached to a metal tip substrate. Due to the strong adhesion, CNTs emit constant currents even after intense arcing events. In addition, CNT emitters can be pre-treated with an electrical conditioning process with the help of strong adhesion, and almost no arcing events are observed during a normal operation.

## Methods

**Figure 1:**
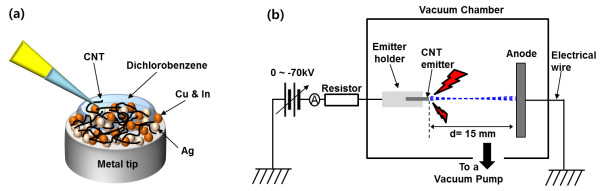
Schematics of the (a) CNT emitter fabrication process and (b) experimental setup for the characterization.

The fabrication process of the CNT emitter is schematically displayed in Figure 1a. The commercial single-walled CNTs (model: CNT SP95, Carbon Nano-material Technology Co., Ltd., Pohang-si, South Korea) were used for the fabrication of CNT emitters. The CNTs were purified using a hydrothermal treatment with a mixture of nitric acid and sulfuric acid for a better CNT dispersion and a complete removal of amorphous carbon [27]. After a CNT solution consisting of 1 wt.% CNT and 99 wt.% 1,2-dichlorobenzene (Sigma-Aldrich, St. Louis, MO, USA) was sonicated at room temperature for 2 h, the CNT solution (3 μl) was mixed with a commercialized metal mixture binder (0.025 g; Premabraze 616, Lucas-Milhaupt, Inc., Cudahy, CA, USA). The metal mixture binder is composed of 61.5 wt.% silver, 24 wt.% copper, and 14.5 wt.% indium micro- and nanoparticles. Metal wires such as copper, kovar, stainless steel (SUS), tungsten, silver, and titanium with a diameter of 1 mm were used as substrates of the emitters. One end of the metal wires was mechanically polished to have a flat surface. Around 0.5 μl of the CNT/metal binder mixture was put on a metal tip substrate. The CNT/metal binder mixture dried out very quickly in approximately 5 min due to high volatility of dichlorobenzene. Subsequently, an annealing process was carried out under vacuum at approximately 10^−6^ Torr at different temperatures. For comparison, a CNT emitter was prepared using silver nanoparticles (NPs; DGH, Advanced Nano Products Co., Ltd., Buyong-myeon, South Korea) under similar conditions.

The morphologies of the fabricated CNT emitters were characterized using a field emission scanning electron microscope (FESEM; Hitachi S-4800, Chiyoda-ku, Japan). The adhesive force of the CNT/metal binder coating on a substrate was measured by a pencil hardness test, which is described in American Society for Testing and Materials (ASTM) D3363. Field emission properties of the fabricated CNT emitters were characterized in a vacuum chamber, which is schematically shown in Figure 1b. A diode type with a copper disc (diameter, 30 mm) acting as an anode was employed for the field emission test. A negative high voltage of 0 ~ −70 kV was applied to the CNT emitter while the Cu anode was grounded. The distance between the CNT emitter and the anode was fixed to 15 mm. In order to protect the high-voltage power supply due to high-voltage arcing, a current-limiting resistor (resistance, 10 MΩ) was installed between the power supply and the emitter.

## Results and discussion

**Figure 2:**
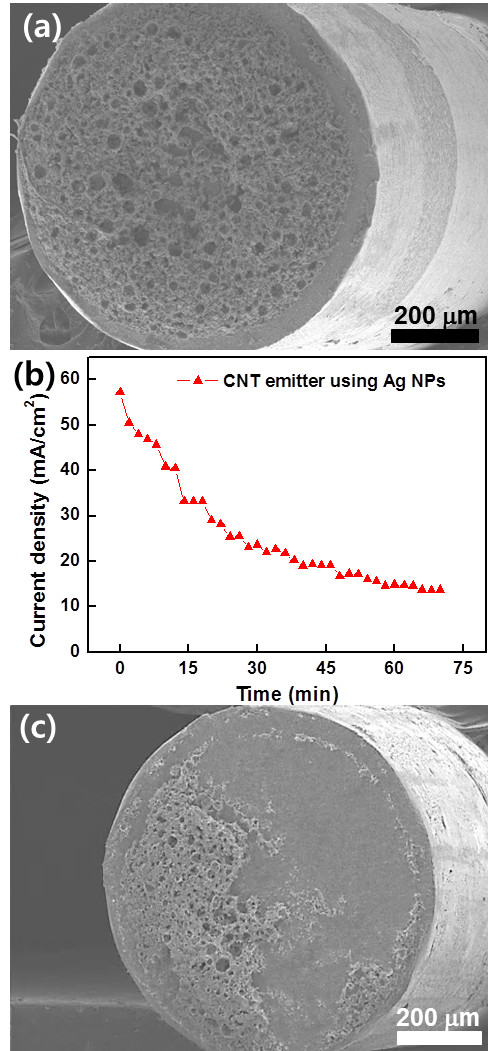
**FESEM images and stability test of the fabricated CNT emitters using silver NPs. (a)** FESEM image of the fabricated CNT emitter using silver NPs on tungsten metal tip. **(b)** Stability test of the fabricated CNT emitter with time. **(c)** FESEM image of the CNT emitter after emission stability experiment. Severe damage of the CNT/silver NP mixture was observed as compared with **(a)**.

The role of metal binders is to attach CNTs to substrates. Silver NPs have been widely used for a metal binder due to good electrical conductivity and good contact with CNTs [3,4,28]. To investigate the performance as a binder, we prepared a CNT emitter on a tungsten metal tip (diameter, 1 mm) using silver NPs (Figure 2a). The annealing temperature to melt silver NPs was 750°C. As shown in Figure 2b, the fabricated CNT emitters exhibited very poor stability. Electron current density emitted from the emitter was initially 57.3 mA/cm^2^ at the applied voltage of 35.5 kV; however, the current density was dramatically reduced to 13.6 mA/cm^2^ for a 70-min operation (Figure 2b). Frequent arcing was observed during the test, and the emission current density was slowly decreased with the increase in the arcing events. A FESEM image clearly shows that approximately 70% of the CNT and silver binder attached on the substrate were removed after the test (Figure 2c). These results indicate that silver NPs could not work as a good binder of a CNT emitter that can withstand against high-voltage arcing. To analyze the bad performance of the CNT emitter, the adhesion force between the silver NP binder and the tungsten substrate was characterized with a pencil hardness test. For the characterization, the silver NPs were annealed on a tungsten sheet (10 × 10 mm^2^) at 750°C. The pencil hardness of the silver film attached to the tungsten sheet was 2B, which is a soft level as determined by ASTM D3363. Such poor adhesion of the silver film might be improved by changing the substrate, and thus, we prepared the silver film on other metal sheets such as SUS, titanium, kovar, and copper. However, the pencil hardness of the silver film did not exceed 1B, reflecting that the adhesive force of the silver binder is not so high on the metal substrates.

**Figure 3:**
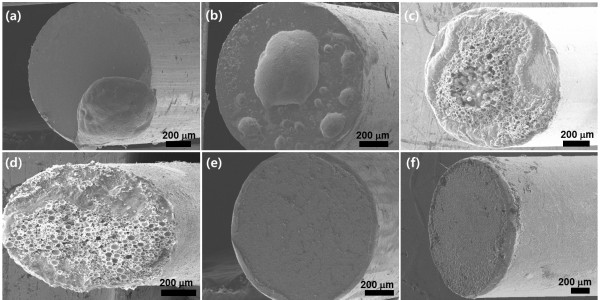
**FESEM images of metal mixture binders on various tip substrates. (a)** Tungsten, **(b)** SUS, **(c)** titanium, **(d)** silver, **(e)** kovar, and **(f)** copper. The annealing temperature was 750°C.

As a candidate of a good binder, we tried to use a brazing filler material that is used to join two different metals. The brazing filler material is a metal mixture composed of silver, copper, and indium micro- and nanoparticles described in the ‘Methods’ section. Before using this material as a binder of the CNT emitters, the adhesion behavior of the material at different substrates was analyzed. As shown in Figure 3a,b,c,d, the metal mixture was melted at 750°C, but the melted metal mixture was spherically aggregated on the tungsten, SUS, titanium, and silver substrates, suggesting a poor wettability to the substrates. However, thin films of metal mixture binders were uniformly formed on kovar and copper substrates (Figure 3e,f, respectively). In addition, pencil hardness tests revealed that the hardness of the metal mixture films on the kovar and copper substrates were 4H. This indicates that the metal mixture films were very strongly attached to the substrate and the adhesive force to the substrate was remarkably enhanced compared to silver NPs.

**Figure 4:**
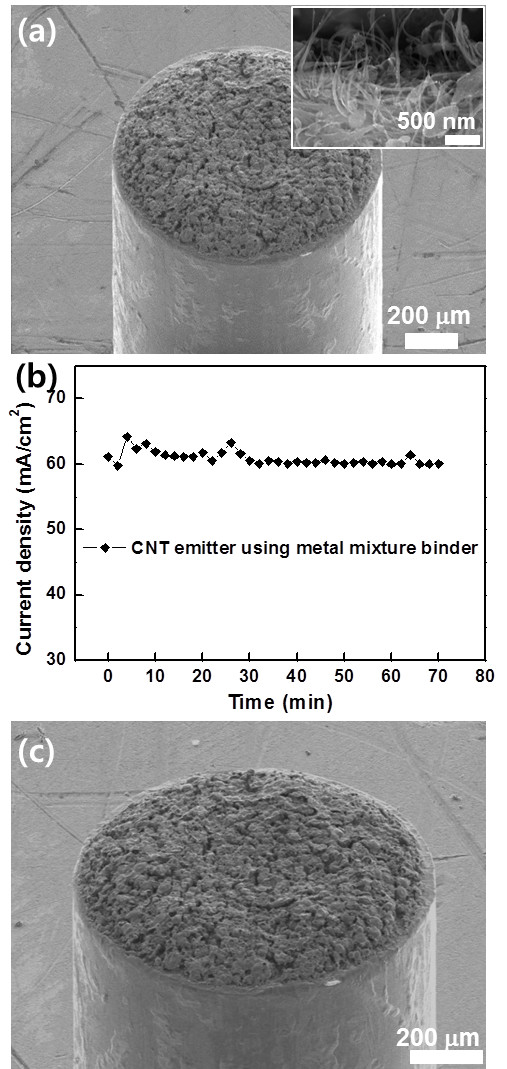
**FESEM images and stability measurement of the fabricated CNT emitters using metal mixture binders. (a)** FESEM image of a CNT/metal mixture binder coated on a kovar tip substrate annealed at 750°C. Inset: vertically standing CNTs formed on the metal tip. **(b)** Stability measurement of the CNT emitter fabricated using the metal mixture binder with time. **(c)** FESEM image of the CNT emitter fabricated using the metal mixture binder after the field emission property measurement.

Based on this fact, CNT emitters were fabricated on kovar and copper tips using the metal mixture as a binder. The metal mixtures were annealed at 750°C. FESEM images of the CNT emitter prepared on a kovar tip show that CNTs were uniformly coated on the kovar tip and vertically aligned CNTs were clearly observed (Figure 4a). Emission current density remained almost constant with time after electrical conditioning, which will be described later (Figure 4b). In addition, even though frequent arcing occurred, the metal binders and the CNTs were still adhered to the tip substrate (Figure 4c). Note that the metal binder and CNTs were seriously detached from the substrate when silver NPs were used as a binder. Therefore, the CNT emitters fabricated using the metal mixture binder exhibited very high stability against arcing.

**Figure 5:**
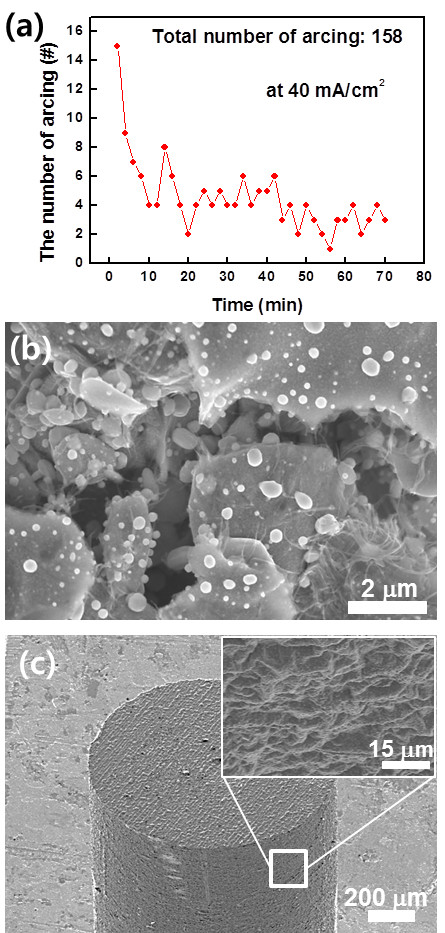
**Number of arcing events and FESEM images of the fabricated CNT emitters on kovar substrates. (a)** The number of arcing events of the CNT emitter fabricated using the metal mixture binder with time at a current density of 40 mA/cm^2^. **(b)** Magnified FESEM image of the CNT/metal mixture binder after field emission tests. **(c)** FESEM image of a CNT/metal mixture binder coated on a kovar metal tip annealed at 900°C (inset: magnified FESEM image of the surface of the kovar substrate).

However, the fact that frequent arcing was observed during the field emission prevents a stable operation of the CNT emitters. As displayed in Figure 5a, approximately 160 arcing events occurred at the emission current density of 40 mA/cm^2^ even after a conditioning process. The reason of such frequent arcing was attributed to non-melted materials in the metal mixture binder. Although it looks like that the metal mixture was melted to form a film on the tip substrate after annealing at 750°C, a FESEM image reveals that some NPs in the mixture were not completely melted and the NPs were exposed to the surface (Figure 5b). Since the non-melted NPs were loosely attached to the binder film, they could be easily detached from the surface by a high electric field [14-16]. When the NPs were detached, an arcing could be induced; the arcing continued until all the loosely bound NPs were completely removed from the surface. This is the reason why frequent arcing events were observed at the CNT emitters. To overcome this problem, the annealing temperature was increased to 900°C. A thin and uniform film of the CNT/metal binder mixture was formed on a kovar tip substrate, and no NPs were observed on the surface because they were completely melted at the temperature of 900°C. However, unfortunately, the surface of the kovar substrate was seriously damaged at the temperature, limiting the practical applications of the CNT emitters (inset of Figure 5c).

**Figure 6:**
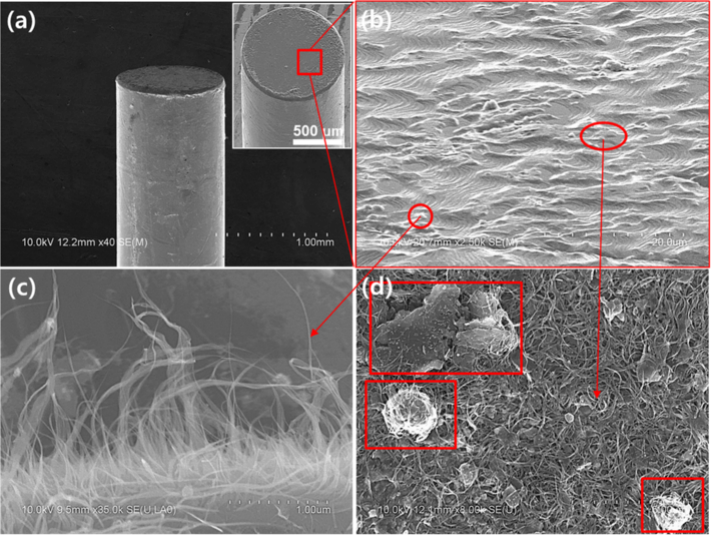
**FESEM images of the fabricated CNT emitter on a copper tip substrate. (a)** FESEM image of a CNT/metal binder coated on a copper tip substrate using the metal mixture binder annealed at 900°C. **(b)** Magnified FESEM image of the CNT/metal mixture binder shown in **(a)**. **(c**, **d)** Magnified FESEM images of the regions marked in **(b)**.

However, the damage of a tip substrate was not observed when copper was used as a substrate. Figure 6 shows the FESEM images of the CNT emitter fabricated on a copper tip. A uniform film of the CNT/metal binder mixture with the thickness of approximately 20 μm was prepared on the copper tip after an annealing process at 900°C (Figure 6a). The magnified FESEM images of the CNT/metal binder mixture (Figure 6b) show that vertically standing CNTs of different heights (Figure 6c) as well as CNTs lying on the side (Figure 6d) were formed on the surface. One end of the vertically standing CNTs was generally embedded in the binder film, suggesting strong adhesion to the coating. In contrast, agglomerates of amorphous carbons or CNTs (rectangular regions in Figure 6d) that were not bound to the coating materials were also observed. The agglomerates of amorphous carbons or CNTs were attributed to an incomplete purification process that was described in the ‘Methods’ section. These agglomerates exert negative effects on the stable operation of the field emitter.

**Figure 7:**
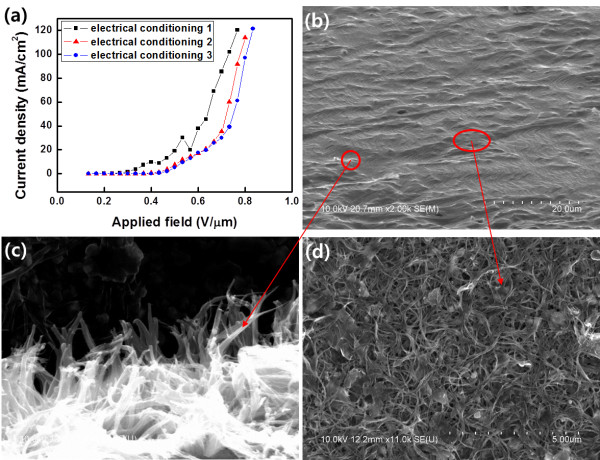
***J*-*E *plots of electrical conditionings and FESEM images of the CNT emitter after conditioning processes. (a)** Typical *J*-*E* plots at different runs of electrical conditioning processes. **(b)** FESEM image of the CNT emitter after conditioning processes. **(c**, **d)** Magnified FESEM images of the regions marked in **(b)**.

In order to remove the loosely bound carbon agglomerates, the as-prepared CNT emitters were treated with electrical conditioning processes [29]. Electrical conditioning is a process to induce arcing intentionally to remove the materials that negatively affect field emission. An electrical conditioning process was carried out by increasing the applied electric field at the emitters by 0.033 V/μm (corresponding to 500 V in these experiments) to 0.83 V/μm (Figure 7a). The electric field at each step was maintained for 5 min, and three runs of the conditioning processes were performed for each CNT field emitter. It should be noted that the electric field (abscissa) shown in Figure 7a was calculated by dividing applied voltage by the emitter-anode distance. However, actual electric fields are much higher than the abscissa values. This is because small metal tips (diameter, 1 mm) were used as the substrates of CNT emitters in our experiments and such small metal tips produce higher electric field than a flat substrate at the same applied voltage [30]. While the electric field was increasing, many arcing events occurred because loosely bound materials on the surface were removed by the strong electric field [14-16]. After three runs of electrical conditioning processes, the loosely bound materials shown in Figure 6d were almost completely removed (Figure 7d). Meanwhile, arcing events inevitably occur during the field emission at emission current densities higher than a critical density of approximately 50 mA/cm^2^[22,23]. This is because emitting CNTs are self-heated due to Joule heating, which can result in a thermal runaway over the critical current density. Due to the thermal runaway, the temperature of CNTs at the tip apex regions increases and accordingly the apex regions can be melted or evaporated. Furthermore, CNTs can be broken at defect sites because electrical resistance at the defect sites is higher than that at other regions, and hence, the temperature can be highly increased at the sites. Since CNTs of greater heights contribute to higher field emission current, thermal runaway is more serious at longer CNTs. As a result, longer CNTs become short [29] and vertically standing CNTs with more uniform heights remained on the substrate after repetitive conditioning processes (Figure 7c). Consequently, through electrical conditioning processes, loosely bound materials on the surface were removed and simultaneously the heights of CNTs became more uniform. During the conditioning process, many arcing events occurred; however, the arcing finally led to more stable field emission because the materials that induce arcing were removed in advance.

**Figure 8:**
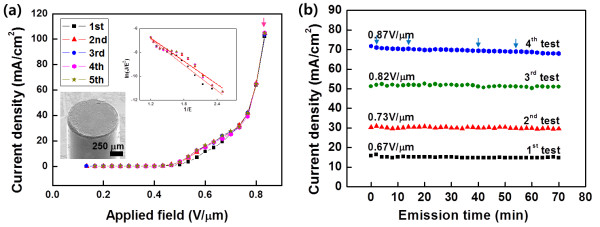
**Field emission properties and emission stabilities of the fabricated CNT emitters after the electrical conditionings. (a)** Field emission properties of the fabricated CNT emitters after the conditioning process. Five *J*-*E* measurements were performed. One arcing occurred at the maximum current density of the fourth run (pink arrow). Inset graph and image in **(a)** are the FN plots of the *J*-*E* curves of the CNT emitter and the wettability of metal mixture binders on the copper tip substrate after annealing at 900°C, respectively. **(b)** Emission stabilities of the fabricated CNT emitters at different electric fields.

Figure 8 shows typical field emission characteristics of the fabricated CNT emitters after the conditioning processes. Current density vs. electric field (*J*-*E*) curves were repeatedly measured. The *J*-*E* curves follow well the Fowler-Nordheim (FN) equation [31] (inset of Figure 8a) with a comparatively high field enhancement factor (*β*) of about 23,000. For comparison, the *J*-*E* curves of the CNT emitters during the conditioning processes were included (Figure 7a). As the conditioning process continued, a threshold electric field corresponding to 10 mA/cm^2^ increased from 0.4 to 0.54 V/μm and the *J*-*E* curves changed. This is because long CNTs become gradually shorter during the conditioning processes and emission current density from each CNT is reduced. However, after the conditioning processes, *J*-*E* curves remain almost constant at the repeated field emission tests (Figure 8a). One thing to note here is that the emission current density reached higher than approximately 100 mA/cm^2^ in the *J*-*E* measurements and a few arcing events occurred at such a high current density. However, in contrast to the conditioning process, the *J*-*E* curves practically do not change even after the arcing events. Figure 8b shows the temporal behavior of the emission current densities at different electric fields, which were measured at a medium vacuum of approximately 10^−5^ Torr. No arcing event occurred at emission current densities lower than 50 mA/cm^2^, and the emission current densities remain almost constant with time. When the current density was increased to 70 mA/cm^2^ that is higher than the critical current density, four arcing events (marked in blue arrows in Figure 8b) occurred for a 70-min operation. However, emission current density does not change after the arcing events, which is clearly shown in Figure 8b. Therefore, the emitters could be operated without arcing below 50 mA/cm^2^ and constant current densities were stably emitted even arcing was induced at higher electric fields, demonstrating that the fabricated CNT emitters exhibit very stable field emission properties. The high stability of the field emitters with high *β* values was attributed to the fact that vertically standing CNTs were strongly attached to the substrates through the metal mixture binder.

## Conclusions

CNT emitters were fabricated on copper tip substrates using a metal mixture that was composed of silver, copper, and indium micro- and nanoparticles as a binder. The metal mixture strongly attached CNTs to the tip substrate. Due to the strong adhesion, CNT emitters could be pre-treated with an electrical conditioning process without seriously damaging the CNTs even though many intense arcing events were induced at the small and sharp geometry of the tip substrate. Impurities that were loosely bound to the substrates were almost removed and CNT heights became uniform after the electrical conditioning process. Consequently, no arcing events were observed from the CNT emitters during the normal operation with the current density less than 50 mA/cm^2^. Moreover, even though arcing was induced at a higher current density of 70 mA/cm^2^, the emitters could withstand the arcing and the emission current remained constant with time. Due to the strong binding of the CNTs to the substrates, CNTs were not detached from the substrates even by the arcing events. Consequently, the fabricated CNT emitters exhibit very stable field emission properties, which are very useful for the realization of miniature X-ray tubes and small-sized electronic devices that require high-voltage operation.

## Competing interests

The authors declare that they have no competing interests.

## Authors' contributions

JMH carried out the design and fabrication of the experimental setups and drafted the manuscript. HJK assisted in the experiments. HSR assisted in the design of the experimental setups. SOC supervised the whole study. All authors read and approved the final manuscript.
